# Emotional reactivity to binge food and erotic cues in women with bulimia nervosa symptoms

**DOI:** 10.1186/s40337-021-00475-9

**Published:** 2021-09-28

**Authors:** Isabel Hernández-Rivero, Jens Blechert, Laura Miccoli, Katharina Naomi Eichin, M. Carmen Fernández-Santaella, Rafael Delgado-Rodríguez

**Affiliations:** 1 Mind, Brain, and Behavior Research Center (CIMCYC), University of Granada, Campus de Cartuja s/n, 18071 Granada, España; 2 Department of Psychology, Centre for Cognitive Neuroscience, Paris-Lodron-University of Salzburg, Hellbrunnerstraße 34, 5020 Salzburg, Austria; 3 Department of Psychology, University of Jaén, Campus Las Lagunillas, 23009 Jaén, España

**Keywords:** Bulimia nervosa, Binge food cues, Erotic cues, Psychophysiology, Valence, Startle reflex

## Abstract

**Background:**

Studies on food cue reactivity have documented that altered responses to high-calorie food are associated with bulimic symptomatology, however, alterations in sexual motivations and behaviors are also associated clinical features in this population, which justify their inclusion as a research target. Here, we study responses to erotic cues—alongside food, neutral and aversive cues—to gain an understanding of specificity to food versus a generalized sensitivity to primary reinforcers.

**Methods:**

We recorded peripheral psychophysiological indices –the startle reflex, zygomaticus, and corrugator responses—and self-reported emotional responses (valence, arousal, and dominance) in 75 women completing the Bulimia Test-Revised (BULIT-R). Multiple regression analysis tested whether BULIT-R symptoms were predicted by self-reported and psychophysiological responses to food versus neutral and erotic versus neutral images.

**Results:**

The results showed that individuals with higher bulimic symptoms were characterized by potentiated eye blink startle response during binge food (vs. neutral images) and more positive valence ratings during erotic (vs. neutral) cues.

**Conclusions:**

The results highlight the negative emotional reactivity of individuals with elevated bulimic symptoms toward food cues, which could be related to the risk of progression to full bulimia nervosa and thereby addressed in prevention efforts. Results also point to the potential role of reactivity to erotic content, at least on a subjective level. Theoretical models of eating disorders should widen their conceptual scope to consider reactivity to a broader spectrum of primary reinforcers, which would have implications for cue exposure-based treatments.

**Plain English summary:**

We examined appetitive and aversive cue responses in college women to investigate how bulimic symptoms relate to primary reinforcers such as food and erotic images. We recorded peripheral psychophysiological indices (the startle reflex, zygomaticus, and corrugator responses) and self-reported emotional responses (valence, arousal, and dominance) in 75 college women that were presented with the Spanish version of the Bulimia Test-Revised. The results showed that bulimic symptoms increase both psychophysiological defensiveness toward food cues and subjective pleasure toward erotic cues. The findings suggest a generalized sensitivity to primary reinforcers in the presence of bulimic symptoms, and emphasize the relevance of adopting a wider framework in research and treatment on bulimia nervosa.

**Supplementary Information:**

The online version contains supplementary material available at 10.1186/s40337-021-00475-9.

## Background

Eating and reproductive behaviors are among the most essential goal-directed behaviors due to their high relevance for human survival. Thus, food-related and erotic cues are generally experienced as motivating and pleasing, reliably engaging the appetitive motivational system [1, 2]. Nevertheless, some psychiatric syndromes, such as bulimia nervosa (BN), which especially affects women [3], tend to deviate from this general pattern, at least for food cues. Patients with this debilitating disorder are characterized by binge eating, inappropriate compensatory behaviors, undue influence of body weight and/or shape on self-esteem [4]. These symptoms appear to be related to altered food cue responses, although it recently emerged that erotic stimuli also tend to be processed differently in patients with BN, at least in terms of subjective emotional reactivity [5, 6].

The scientific literature has documented the presence of altered food cue reactivity in women with BN [7–10]. According to studies examining peripheral and subjective reactivity to food cues, high-calorie food pictures are processed as moderately pleasant and activating by healthy women [1, 2], however, the same pictures evoke affective ambivalence in women with BN and individuals with binge eating, since their urge to eat is accompanied by anxiety and fear of weight gain [7]. This ambivalence takes the form of a discrepancy between psychophysiological responses—that show enhancement of the corrugator supercilii muscle activity and the eye-blink startle reflex, both indicating aversive responses—, and subjective responses—that tend to indicate appetitive food processing [11, 12].

Previous studies that analyzed peripheral psychophysiological responses to food stimuli in BN patients and women with subclinical bulimic symptoms have used standardized food cues, including high-calorie foods [11–13]. However, not all foods have the same emotional meaning for people with food-related problems [14, 15], since binge-food preferences are highly idiosyncratic [16, 17]. Thus, the present study individually selected binge food images for each participant (see also [6, 16]. Exploring subjective and psychophysiological reactivity to specific binge food cues has implications for clinical practice, since binge foods are the cue target of exposure therapy [18–20], through which BN patients can develop a more adaptive emotional pattern and prevent binge-purge cycles [21].

Although, based on the fifth edition of the Diagnostic and Statistical Manual of Mental Disorders (DSM-5; [4], sexual dysfunction is not a core symptom of eating disorders, a body of clinical and empirical literature has linked BN with sexuality and intimacy dysfunctions [22, 23]. Some authors have suggested similarities between eating and sexual behaviors in women with BN, stressing that eating more food than the normal population, but feeling more discomfort associated with that intake, is paralleled by their sexual behavior [24]: women with BN tend to show greater sexual activity [23, 25] and interest in erotic cues compared to healthy women [26], although, at the same time, they experience greater sexual dissatisfaction and lower sexual self-esteem [23, 27]. Sexual dysfunctions are associated with different forms of compensatory behaviors [28], which, according to the authors, might be used by bulimic women to achieve their body ideal and feel more comfortable during sexual intimacy. Despite the relationship between BN and intimacy and sexuality issues, research on this topic has been limited to clinical observations and self-reports [23, 29, 30]. To the best of our knowledge, only one study specifically used erotic pictures to explore BN subjective reactions [5] and found that women with BN subjectively report less pleasure and greater dyscontrol than healthy participants. Recently, another study provided objective evidence that women with BN process food and erotic cues differently: Delgado-Rodríguez and colleagues [6] investigated emotional brain responses in BN patients and showed that pictures of personal binge foods and erotic couples were accompanied by a larger late positive potential (LPP) in full-diagnosis BN patients, whereas patients did not differ from controls in their reactivity to unpleasant and neutral cues. However, the LPP is primarily sensitive to emotional arousal; it increases with the motivational salience of the stimuli, regardless their positive or negative valence [31], whereas other psychophysiological measures are sensitive to the appetitive or aversive nature of the stimuli.

The present study follows up on Delgado-Rodríguez and colleagues’ findings by examining the motivational significance of food and erotic cues in a nonclinical sample of women with bulimic symptoms. To characterize the valence dimension, we examined peripheral indicators, such as corrugator and zygomatic muscle activity together with the eye-blink startle, that are sensitive to the activity of both the aversive and the appetitive motivational systems [1]. In addition, affective ratings of valence, arousal, and dominance were also evaluated using the Self-Assessment Manikin (SAM; [32]. We examined a sample of college women with bulimic symptoms (a) to evaluate whether altered cue reactivity to food and erotic images are visible also at early subclinical stages of the disorder and (b) to explore the dimensional characteristics of such cue reactivity. Disordered eating patterns tend to emerge early, during adolescence [33], with the result that they are very common among university women [34, 35]. It is therefore relevant to investigate eating disorders within nonclinical samples of college women because they may still benefit from broad preventive programs aimed at the early stages of food-related disorders [34, 36]. From a more theoretical perspective, confirming that subclinical BN symptoms are also accompanied by altered reactivity to food and body-related cues would further support the necessity of such prevention programs and help refining their target. Previous findings of altered response to high-calorie food cues in adolescents with risky dieting practices [37] and in women with binge-purge eating patterns [11] suggest as a whole that food processing disturbances are present also at subclinical levels of the disorder. However, erotic cue processing has not been previously investigated in nonclinical women with bulimic symptoms. Thus, to sum up, the present study aims to examine, in college women, how bulimic symptoms relate to appetitive and aversive cue responses across a wide spectrum of stimuli, both related and unrelated to binge food and erotica. Our ultimate goal is to understand BN cue reactivity within a broader frame of reference.

## Methods

### Participants

A total of 82 healthy women from the University of Granada participated in this study between November 2018 and April 2019. We excluded women who reported pregnancy; past and present drug abuse; a psychotic disorder; visual, auditory, cardiovascular, or neurological diseases; or current or past treatment for eating or weight-related disorders. Female university students have high rates of eating disorders problems [34], therefore the sample was considered to be adequate and representative for studying the association between BN symptoms and emotional reactivity to binge food and erotic cues. Some participants were excluded due to technical problems with the startle probe (n = 2), corrugator electromyographic (EMG) (n = 1), and body mass index (BMI) measure (n = 2), for suffering from blood phobia (n = 1), or for being in a fasting state at the experiment onset (n = 1). Thus, complete data were available for 75 participants.

### Stimuli

The participants viewed a total of 96 pictures belonging to 4 different categories: unpleasant (n = 24) and neutral (n = 24) pictures were taken from the International Affective Picture System (IAPS)[38],^1^ and erotic pictures were taken from both the IAPS (n = 19) and the Erotic subset of the Nencki Affective Picture System (NAPS ERO; [39]^2^ (n = 5). The pictures were taken from two different sources in order to avoid selecting more explicit sexual content (e.g., opposite-sex erotica) which in women, although subjectively perceived as pleasant and activating, also prompt psychophysiological reactions that indicate the activation of the aversive system, i.e., increase in corrugator muscle activity and startle reflex [2]. Therefore, all selected erotic pictures depicted erotic couples: romantic couples during physical intimacy, either partially unclothed or naked (i.e., non-explicit erotic cues), in order to avoid women’s mixed motivation toward more sexually explicit pictures. Indeed, pictures of erotic couples prompt women’s greatest startle probe inhibition, indicative of highly appetitive motivation [2].

Food pictures were individually selected for each participant based on foods they “found personally irresistible and that they could keep eating until binging”. It has been shown that personally significant stimuli, such as faces of loved ones or cues of personal phobias, are associated with high levels of physiological reactivity [40, 41]. In our previous study [6], the presentation of personal binge food cues successfully prompted subjective feelings of arousal that were higher than those observed using nonpersonal high-calorie food cues. Thus, during a telephone interview, participants reported their six personal binge foods, describing them meticulously and giving at least two descriptors of each food (e.g., lasagna with meat, cheese, and bechamel). Foods had to be high in calories, and could be sweet as well as salty. Based on each participant’s description, the experimenter searched the Internet for four different images of each personal binge food (n = 24), therefore, participants did not see them before the experiment. Picture selection followed the same rationale as in previous studies [42, 43]: selected food pictures had a high digital resolution and displayed food on a naturalistic, nonuniform background that corresponded with the perceptual properties of nonfood IAPS pictures. Even though our selection procedure could lead to select food images differing in perceptual features from the IAPS images, it should not affect results since previous emotional literature indicates that perceptual features as color, contrast, spatial frequency, and complexity does not influence peripheral reactivity [1, 44].

### Apparatus and physiological measures

Signal acquisition and stimulus presentation were carried out with a PC running VPM software, v.12.6 [45]. Startle reflex and EMG activity of the zygomaticus major and corrugator supercilii muscles were recorded through a Coulbourn polygraph, LabLink V model (Coulbourn Instruments, Lehigh Valley, PA), using three V75-04 bioamplifiers with miniature electrodes filled with conductive gel. For startle responses, following Blumenthal and colls.’ guidelines [46], startle probes were delivered through headphones (model Telephonics TDH49P) by a Coulbourn S81‐02 white noise generator. A Brüel & Kjaer sonometer (model 2235) and an artificial ear (model 4153) were used to calibrate the noise intensity. The raw EMG signals were bandpass-filtered (28–500 Hz) and transformed into integrated EMG in μV with a V76-24 integrator. The time constant for the zygomaticus and corrugator was 500 ms, with a sampling rate of 100 Hz, whereas the time constant for the orbicularis was 20 ms with a sampling rate of 1000 Hz. Skin conductance and electroencephalography were also recorded; however, these measures were not included in the present paper.

### Self-report measures

The *Bulimia Test-Revised* (BULIT-R, 47) is a self-report measure of BN symptoms that consists of 36 items that, in the Spanish version, covers four main factors –body concerns, binge, diuretics, and laxatives—[48]. This test was designed to detect the risk of suffering from BN in the general population. In Thelen et al.’s [47] study, scores of ≥ 104 were associated with a BN diagnosis, though they proposed a score of ≥ 85 as an alternative cut-off to reduce the number of false negatives. In the present study, internal consistency was high (α = .90), similar to the results reported by Thelen et al. [47] (α = .92–.98; 1991). The Spanish version of the BULIT-R has reliable psychometric properties in college students [48].

The *Self-Assessment Manikin* (SAM; [32]) is a nonverbal pictorial rating scale often used along with the IAPS [38] to rate the valence (pleasant vs. unpleasant), arousal (relaxed vs. activated), and dominance (feeling in control vs. feeling controlled) elicited by images. Nine intensity levels are represented by 5 humanoid figures and the spaces between these figures. The SAM has been widely validated and is extensively used in cue reactivity research [49, 50]. It has demonstrated a strong consistency in the covariation between physiological responses and self-reports of valence and arousal [32].

### Procedure

The participants were contacted at their lecture classroom where the experiment was introduced. Women who decided to participate were later interviewed by phone. In this interview, the experimenter checked that participants did not meet the exclusion criteria and gathered information about the six personal binge foods that they could “eat and eat until binging” (see Stimuli). Eligible participants were invited to two experimental sessions at the laboratory.

In the first session, they signed an informed consent form and completed the BULIT-R questionnaire [48]. To obtain a broader understanding of the sample population, other ED characteristics were also assessed using the Eating Disorder Examination Questionnaire (EDE‐Q) ([51]; Spanish version by [52]), the Yale Food Addiction Scale (YFAS 2.0) ([53]; Spanish version by [54]), the Snaith-Hamilton Pleasure Scale (SHAPS) ([55]; Spanish version by [56]), and the Body Shape Questionnaire (BSQ) [57, 58]. The participants’ BMI was estimated using a Leicester height measure stadiometer recorded to the nearest millimeter and an electronic body composition analyzer (Tanita Model 300MA, Chicago, IL). Participants were then invited to a second session for the psychophysiological recording and were made aware of the testing requirements for this session: not having their period, not taking any medication, not consuming drugs or alcoholic beverages, and avoiding excessive exercise 24 h before the session. To avoid food deprivation and to match the hunger level between participants, they were instructed to eat a piece of toasted bread with olive oil or butter (between 400 and 415 kcal) without eating or drinking anything but water, 2.5 h before the physiological recording.

The second session was devoted to the psychophysiological data recording during the affective picture viewing and took place one week after the first session, either in the morning (8:30 AM–12:30 PM) or in the afternoon (3:00–5:00 PM). Upon arrival at the laboratory, a glucose test assessed whether participants had adhered to the instructions of having a small snack. After sensor and headphone placement, their hunger level was assessed. A 19″ flat screen, located 60 cm from the participants, was used to present the pictures. Picture delivery was controlled by a computer running Presentation (v.16.3, Neurobehavioral Systems, San Francisco, CA). Participants were asked to view each picture the entire time that it was on the screen. Four pseudorandomized picture orders were used across the participants. Each order included 96 picture trials, avoiding more than two repetitions of the same picture category. After a five-minute baseline, the 96 picture trials were displayed, each comprising 4 s of baseline and 6 s of picture viewing, followed by a variable intertrial interval (ITI) of 10–22 s. The startle probes consisted of 50 ms white noise bursts (105 dB) presented through headphones during 75% of the trials after 4, 4.5, 5, or 5.5 s from picture onset. To decrease their predictability, the probes were also randomly delivered during 1/8 of the ITIs.

After the picture viewing, once the sensors and headphones were removed, the women reported their hunger level and rated all pictures on the SAM scales of valence, arousal, and dominance [32]. After an explanation about the purpose of the study, the participants were thanked and compensated for their time with course credits.

### Data reduction and statistical analysis

To define the startle reflex amplitude, we used the difference in microvolts between the peak and the onset of the response in a time window between 20 and 120 ms after stimulus onset, using the algorithm described by Balaban and colls [59]. Corrugator and zygomatic EMGs were scored as the mean activity during the first 4 s of picture presentation, before startle probe delivery. To remove between-subject variability, startle amplitudes and corrugator and zygomatic EMG responses to each picture category were *t*-score standardized (mean = 50; SD = 10) using the individual mean across all picture categories.

We performed a hierarchical linear regression to examine whether psychophysiological and subjective reactions to food and erotic pictures predicted the presence of bulimic symptoms. Following previous literature [60–63], for startle reflex, corrugator and zygomatic muscle reactivity, and for valence, arousal, and dominance ratings, the difference in reactivity to food minus neutral cues, and to erotic minus neutral cues were introduced as predictors. Thus, we assessed whether the baseline-corrected amplitude of each woman’s reaction to food and erotic cues predicted the amount of bulimic symptoms she reported. Predictor scores greater or less than 3 SD were winsorized to ensure that distributions were less sensitive to outliers while maintaining statistical power. A small number of self-reported and psychophysiological responses were winsorized (corrugator: 4; zygomatic: 1; valence: 1).

In order to select the predictors to be included into the multiple linear regression analysis, we ran, in a first step, Pearson’s bivariate correlations between each of the psychophysiological and self-reported measures (food vs. neutral and erotic vs. neutral) on the one hand and the BULIT-R score on the other. Only measures with significant correlations with the BULIT-R score were included into the following two-step hierarchical linear regression analysis predicting BULIT-R scores. Based on its relevance in food-related disorders [64], BMI was also entered into the regression analysis (at the first step), along with psychophysiological and self-report predictors (at the second step). Significance was set at 0.05.

## Results

### Descriptive statistics

Descriptive statistics for the BULIT-R, EDE-Q Global and subscale scores, BSQ, YFAS, and BMI are presented in Table 1. To get an overview of the degree of clinical eating disorder symptomatology, we examined the participants according to BULIT-R and EDE-Q established cutoffs. In the sample, 7.7% (n = 6) passed the BULIT-R cut-off for BN diagnosis (i.e., 85; [47]), and 21% (n = 16) had an EDE-Q Global score above 2.8, which falls into the 85th percentile of Spanish normative EDE-Q data [52]. Breaking down behavioral symptoms of BN, 19% of women (n = 15) endorsed at least one objective binge eating episode in the past month and reported a mean of 2.4 binge eating episodes during the last month; 40% (n = 31) engaged in at least one compensatory behavior to control weight and informed of 4.8 inappropriate eating behaviors in the last month. Approximately one third of the sample (31%, n = 24) reported high body dissatisfaction (scores above 105; [58]), the most potent predictor for eating disorders [65]. Regarding food addiction, a food problem that seems to overlap with bulimic symptoms even in individuals with no clinical diagnoses of disordered eating [66, 67], 10.3% of women (n = 8) could be classified as food addicted. Notwithstanding the mean BMI of the whole sample (20.06) indicating normal-weight, five women were underweight, 16 overweight, and 2 obese.

**Table 1:** Participants’ characteristics

*Demographics*	
Age	20.06(2.78)
Body mass index (kg/m^2^)	22.54(3.54)
Blood glucose (mg/dL)	81.04(27.90)
*Questionnaires*	
BULIT-R	56.03(16.083)
SHAPS	20.79(4.54)
EDE-Q	1.51(1.22)
Bingeing	0.46(1.10)
Compensatory behaviours	1.90(4.76)
BSQ	82.31(33.04)
YFAS	1.35(2.42)
Hunger:	
Level of hunger pre psychophysiological registration^1^	4.30(1.77)
Level of hunger post psychophysiological registration^1^	6.26(1.88)
*Are you hungry? (yes/no)*	35/43
How strong is your desire to eat?^1^	4.13(2.09)
How full do you feel?^1^	3.94(1.91)
How much food do you think you could eat?^1^	5.18(1.77)
How long since your last meal? (in min)	214(24.8)

Values are presented as the means and standard deviations

BULIT-R = Bulimia Test Revised; SHAPS = Snaith-Hamilton Pleasure Scale; EDE-Q: Eating Disorder Questionnaire; BSQ = Body Shape Questionnaire; YFAS = Yale Food Addiction Scale

^1^Questions on hunger level were measured on a 1–9 Likert scale

### Food and erotic cues reactivity as predictors of Bulimic Symptoms

**Fig. 1:**
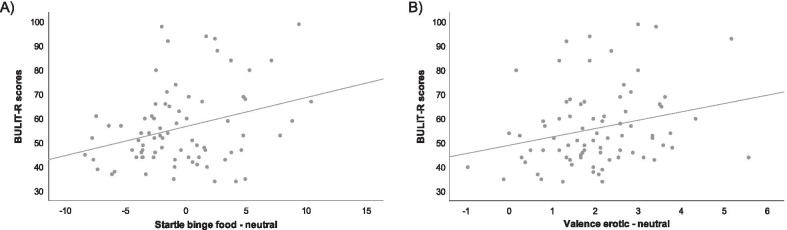
Graphical representation of the Pearson’s bivariate correlations. **a** Represents Pearson’s bivariate correlations between BULIT-R scores and Startle binge food versus neutral and **b** represents Pearson’s bivariate correlations between BULIT-R scores and Valence erotic versus neutral

Table 2 displays the results of Pearson’s bivariate correlations between all psychophysiological and self-reported measures and the BULIT-R scores; only the three measures that significantly correlated with bulimic symptoms were included into the multiple linear regression model (see Fig. 1): BMI; for startle blink amplitude, the difference between food and neutral cues; and, for valence ratings, the difference between erotic and neutral cues.

**Table 2:** Pearson’s bivariate correlations between psychophysiological and self-reported measures and BULIT-R total score

	Hunger	BMI	Binge food-neutral					Erotic-neutral				
			Valence	Arousal	Zygomatic	Corrugator	Startle	Valence	Arousal	Zygomatic	Corrugator	Startle
BULIT-R	− .14	.30*	.11	− .02	.20	.05	.31**	.25*	.05	.12	.11	.16

BULIT-R, Bulimia Test Revised; BMI, Body Mass Index. Hunger refers to level of hunger pre psychophysiological registration

^*^p < .05

^**^p < .01

The second model resulting from the hierarchical regression analyses predicted 23.9% of the variance in BULIT-R scores (Table 3). In line with linear correlations, BULIT-R scores were positively associated with BMI, Startle food-neutral, and Valence erotic-neutral: Greater startle reflex amplitudes when viewing food compared to neutral images—indicative of defensive processing during food cues—were associated with more bulimic symptoms. Moreover, more positive valence responses when viewing erotic vs. neutral images–indicative of appetitive processing during erotic cues—were also associated with more bulimic symptoms. Finally, consistent with literature [64], BMI was a significant predictor of BULIT-R scores, so that having a higher BMI was related to more bulimic symptoms. Although based on Pearson’ bivariate correlations hunger was not related to BULIT-R scores, we run an additional hierarchical regression that included all predictors from Table 3 and hunger level (reported right before the psychophysiological task) to control for the possible confounding impact of hunger on the results. The data did not change.

**Table 3:** Hierarchical linear regression predicting BULIT-R scores

Predictors	Standardized B	Adj. R^2^
*Model 1*		.07*
BMI	.29*	
*Model 2*		.239***
BMI	.30**	
Startle binge food-neutral	.35**	
Valence erotic-neutral	.29**	

BULIT-R, Bulimia Test Revised; BMI, body mass index; Adj. R^2^, adjusted R^2^. ΔR^2^ for model 2 = .19

^*^p < .05

^**^p < .01

^***^p < .001

A post hoc power analysis was conducted using the software G*Power 3.1 [68]. A sample size of 75 was used for the statistical power analyses with three predictor variables, an alpha value of 0.05, and an observed R^2^ of 0.27 (*f*^*2*^ = 0.37) for the whole regression model. The post hoc analyses showed an observed statistical power of 0.99 for this multiple regression study.

## Discussion

The current study examined the peripheral psychophysiological and subjective emotional cue reactivity to two classes of primary reinforcers with relevance to eating disorders—i.e., binge food and erotic cues—, together with neutral and unpleasant stimuli, in a nonclinical sample of college women varying on bulimic symptoms. In line with literature, BMI significantly correlated with bulimic symptoms. Moreover, the findings also revealed that bulimic symptoms were associated with a defensive emotional reactivity to binge food cues—potentiation of the startle reflex—and an exaggerated hedonic processing of erotic cues—high valence responses. Taken together, the data point out that the presence of bulimic symptoms at subclinical stage of BN are accompanied by both aversive psychophysiological reactivity to food cues and high subjective pleasure towards erotic cues, overall suggesting the necessity to approach a wider framework for BN research and treatment.

### Aversive reactivity towards binge food pictures as a predictor of bulimic symptoms

Previous research examining standardized food stimulus processing in women with subthreshold [11] and full syndrome BN [12] showed aversive reactions to standardized food cues: a significant potentiation of startle blink magnitudes. Moreover, in contrast to the startle reflex findings, both Drobes and Mauler found that in women with disordered eating self-reported valence was consistent with an appetitive reaction to nonpersonal food cues. This divergent pattern between psychophysiological and subjective responses led to the hypothesis that in this population food cues might prompt a state of motivational ambivalence [11, 12, 69]. Interestingly, the opposite pattern of motivational conflict has also been observed [13]: BN females had attenuated (i.e., appetitive) startle responsiveness along with aversive subjective responses to food cues. However, in Friederich and colls.’ [13] study there was no control of food deprivation, a key variable that modulates emotional responses to food cues [11, 70]. Our findings on nonclinical women with BN symptoms extend past research [11]: based on psychophysiological data, the greater presence of bulimic symptoms is associated with negative processing of binge foods, visible in enhanced startle potentiation; however, subjective data do not indicate affective ambivalence, as valence does not suggest exaggerated appetitive processing of binge food cues.

Discrepant findings may be due to differences in sample characteristics: while BN-like participants from Drobes et al.’s study [11] were selected according to scores on binge-purge and dietary restraint items (“subjects who scored in the upper 10% on binge-purge items and in the bottom two-thirds on dietary restraint items”, p. 167), for participants’ selection we used the BULIT-R scores that not only include binge and inappropriate compensatory behaviors, but also body-concerns items [48]. In this regard, Racine et al. [60], likewise examining the peripheral psychophysiology during food cues in a nonclinical sample, observed that binge eating episodes and eating disorder cognitions (especially weight and shape concerns) were associated with different emotional patterns: while binge eating was associated with motivational ambivalence, eating disorder cognitions were associated with aversive psychophysiological reactivity to food cues. We hypothesize therefore that in the present research the low number of binge eating episodes (87% of women who binge reported less than 4 episodes during the last month), together with the fact that BULIT-R scores reflect also body-concerns, might have biased the results toward an aversive processing of food cues. Accordingly, we expect that a greater presence of binge eating symptoms might have led to an ambivalent emotional response to food cues.

Another possible explanation is that in women with more bulimic symptoms personalized binge food pictures might have potentiated the threat to control over food cues, again biasing the results toward aversive processing. Participants in the current study were presented with the “specific foods that they found irresistible and that they could keep eating until binging”, thus, in women with bulimic traits, these foods are capable of prompting the irresistible cravings associated with binge onset [71]. That is to say, in some individuals psychophysiologically aversive reactions might be generated by idiosyncratic binge food cues: aversion and defensiveness might emerge as a result of the need to avoid cues that are perceived as a threat.

During the food pictures selection procedure (in which participants were asked to describe their six high-calorie irresistible foods), we did not instruct participants to balance individual foods on regard to food content (sweet vs. salty), thus, the number of both food contents differ between participants. Extra analyses (see Additional file 1) indicated that food contents did not influence emotional reactivity. We highlight the relevance of this procedure because it allows to select the most irresistible food (i.e., asking participants to select sweet and fat foods [e.g., 3 sweet and 3 fat foods] might result in not selecting the most irresistible foods). Then, this procedure allows to explore the emotional reactivity toward emotionally charged foods, which has implications for clinical practice since binge foods are the cue targets of exposure treatments [18–20] that aim to generate more adaptive emotional patterns to prevent binge-purge cycles [21].

### Elevated subjective reactivity to erotic pictures as a predictor of bulimic symptoms

Sexual dysfunction is not a diagnostic criterion for BN; however, previous literature links BN symptoms with sexuality and intimacy difficulties, and points to these problems as influencing factors in the development and maintenance of the disorder [23, 72, 73]. Clinical observations and self-report studies have hypothesized that, in women with BN, sexual behaviors may resemble their food cue reactivity in terms of a marked ambivalence. Individuals suffering from BN, compared to healthy women, tend to have more sexual activity and sexual partners, greater sexual desire and fantasies [23, 73, 74], and more impulsive sexual profiles [22, 75]; however, they also experience increased performance pressure [25, 27], less sense of control [5], and high levels of overall sexual dissatisfaction [23, 73]. Being more sexually active but feeling more dissatisfied might be comparable to their eating behavior; women with BN consume more food during binges than healthy women, but feel greater discomfort associated with their food intake [23].

Findings from the current study did not show negative feelings during sexual pictures: bulimic symptomatology was associated with heightened subjective pleasure responses toward erotic couple pictures. Conversely, previous research on self-reported emotions to more heterogeneous erotic contents (i.e., erotic couples and opposite-sex erotica) found that women with full-diagnosis BN felt less pleasure and greater dyscontrol than healthy women, which, according to the authors, supported BN coactivation of aversive and appetitive mechanisms when processing erotic pictures [5]. Two hypotheses might be suggested to explain such discrepancy. The first one proposes that although erotic cues might prompt increased pleasure at an early, subclinical level, the worsening of such symptoms into full BN may lead to affective ambivalence, similar to the mixed motivation observed with food stimuli [12, 13]. Alternatively, divergence in valence ratings might be due to stimuli selection: in healthy women, erotic couples (i.e., nonexplicit erotic content) tend to provoke appetitive subjective and psychophysiological processing, whereas more explicit sexual contents (i.e., opposite-sex erotica) tend to prompt more aversive reactions, visible in women’s increased corrugator and startle reflex [2]. Future studies might further investigate how different erotic contents are processed in subclinical and full-diagnosis BN in order to elucidate whether explicit and non-explicit erotic contents provoke different emotional reactions or, rather, whether the worsening of the disorder disrupts the affective processing of erotic cues.

Another relevant aspect related to erotic cues is that naked or semi-naked female bodies appear in these images. Body cues (i.e., pictures depicting other’s female body or body parts) are differently processed by women with eating-related problems, specifically, women with BN subjectively assess ‘idealized, thin’ female bodies as highly anxious [13]. Although women appearing in erotic cues (as used in the current study) mostly have idealized bodies, female bodies are not determining the association between bulimic scores and valence ratings (reported in Table 3). Erotic cues reflect a sexual/intimate environment depicting a woman sexually interacting with a naked or semi-naked man; conversely, body cues only depict female body or body parts (typically without head or face; e.g., [13, 76]). Moreover, female bodies or body parts depicted in body cues are clearly presented to facilitate the participants’ body comparison (e.g., wearing bikinis or fitted sports clothing to highlight body shape) (e.g., [76–78], however, erotic cues not always fulfill this assumption (e.g., some women are dressed with clothes that do not highlight their shape). Despite the above-mentioned differences between erotic and body cues, we performed extra analyses to examine the possible impact of female bodies on the current valence result (i.e., the association between valence reactions to erotic cues and bulimic symptoms). Additional analyses (see Additional file 1) showed that valence ratings did not depend on how female bodies appeared in the pictures, but only on BULIT-R scores. Although the influence of female bodies does not seem to be critical for the current results, we consider that future studies might benefit from controlling if women with BN symptoms are influenced by female bodies while viewing erotic cues. Recording objective measures to identify the time and gaze pattern (e.g., using an eye tracker) while viewing erotic cues might allow to have a deeper understanding of erotic cue processing in BN.

The implications of the current results should be evaluated while considering some methodological limitations. The primary limitations are related to our sample selection. Recruiting an unselected sample of undergraduate women resulted in only six participants exceeding BULIT-R cut-off for BN; increasing the number of women exceeding this cut-off might uncover differences in psychophysiological reactivity to erotic cues between healthy and subclinical BN women. Moreover, the use of a diagnostic interview (e.g., EDE; [79]) should be used instead of questionnaires to select a subclinical or clinical sample of BN. Another limitation, which is common in eating disorder studies, is that the sample is limited to women, which might raise concerns that these findings can be generalized to men with BN. Lastly, it should be considered that most of the previous research used to discuss results from food and erotic cue reactivity referred to women with a full diagnosis of BN, while our results are based on a nonclinical sample of women with bulimic symptoms.

The current study is the first that examines peripheral psychophysiological responses in a wider field of relevant stimuli for women with bulimic symptoms. This is remarkable because the use of cues other than food helps to understand the complexity of binge-purge symptomatology. In this sense, although scarce, previous research that used non-eating disorder-related stimuli (e.g., facial expression images or tones in a tone discrimination task) has found that individuals with BN may exhibit impaired cognitive processing across different stimulus classes [9, 80–82]. Another strength is that food cues are highly individualized; using personalized—emotionally charged—binge foods to measure emotional responses to cues directly related to altered behavioral responses in BN.

## Conclusions

The current findings highlight that in a nonclinical sample of women, bulimic symptoms are accompanied by altered peripheral psychophysiology—increased startle reflex—during idiosyncratic binge food cues, indicating the early appearance of defensive reactions to food cues in correspondence with bulimic symptomatology. Moreover, rating data point out that the presence of BN symptoms is accompanied by the early appearance of alterations in the processing—increased subjective pleasure—during cues depicting sexual intimacy. Therefore, early BN symptoms increase both psychophysiological defensiveness toward food cues and subjective pleasure during erotic cues. Interestingly, as mentioned above, it has been observed that full-diagnosis BN is accompanied by larger brain potentials (LPP, indicative of motivational relevance) during both binge food and erotic cues [6], overall suggesting the late emergence of BN psychophysiological sensitivity to both food and erotic cues. Taken as a whole, the data emphasize the relevance of adopting a wider framework in research and treatment on BN, encompassing a broader spectrum of primary reinforcers.

## Supplementary Information

**Additional file 1**. Extra analyses to examine the influence of food contents on emotional reactivity and the influence of female bodies on valence results.

## Data Availability

The datasets used and/or analysed during the current study are available from the corresponding author on reasonable request.

## Abbreviations

APA: American Psychiatric Association
BMI: Body mass index
BN: Bulimia nervosa
BSQ: Body shape questionnaire
BULIT-R: Bulimia test-revised
EDE‐Q: Eating disorder examination questionnaire
EMG: Electromyographic
IAPS: International affective picture system
LPP: Late positive potential
NAPS: Nencki affective picture system
SAM: Self-assessment Manikin
SHAPS: Snaith-Hamilton pleasure scale
YFAS: Yale food addiction scale

## Footnotes

1 IAPS codes for unpleasant: 1525, 2683, 2688, 2800, 2981, 3110, 3530, 6200, 6313, 6315, 6350, 6415, 6510, 6540, 6550, 6555, 6570, 8485, 9075, 9163, 9410, 9630, 9635, 9810; IAPS codes for neutral: 5531, 7000, 7002, 7004, 7009, 7010, 7012, 7020, 7025, 7035, 7040, 7045, 7055, 7059, 7080, 7090, 7150, 7175, 7185, 7224, 7233, 7235, 7705, 7950); IAPS codes for erotic: 4604, 4607, 4608, 4623, 4643, 4651, 4652, 4653, 4658, 4659, 4668, 4669, 4670, 4672, 4676, 4680, 4687, 4695, 4697).

2 NAPS codes for erotic: Opposite-sex_couple_011_h, Opposite-sex_couple_ 015_h, Opposite-sex_couple_016_v, Opposite-sex_couple_017_v, and Opposite- sex_couple_020_h

## References

[1] Bradley MM, Codispoti M, Cuthbert BN, Lang PJ (2001). Emotion and motivation I: defensive and appetitive reactions in picture processing. Emotion.

[2] Bradley MM, Codispoti M, Sabatinelli D, Lang PJ (2001). Emotion and motivation II: sex differences in picture processing. Emotion.

[3] Keski-Rahkonen A, Mustelin L (2016). Epidemiology of eating disorders in Europe: prevalence, incidence, comorbidity, course, consequences, and risk factors. Curr Opin Psychiatr.

[4] American Psychiatric Association (2013). Diagnostic and statistical manual of mental disorders.

[5] Rodríguez S, Mata JL, Lameiras M, Fernández MC, Vila J (2007). Dyscontrol evoked by erotic and food images in women with bulimia nervosa. Eur Eat Disord Rev.

[6] Delgado-Rodríguez R, Hernández-Rivero I, Fernández-Santaella MC, Vila J, Guerra P, Miccoli L (2019). Neural processing of food and erotic cues in bulimia nervosa. Psychosom Med.

[7] Giel KE, Teufel M, Friederich H, Hautzinger M, Enck P (2011). Processing of pictorial food stimuli in patients with eating disorder-A systematic review. Int J Eat Disord.

[8] Chami R, Cardi V, Lautarescu A, Mallorquí-Bagué N, McLoughlin G (2019). Neural responses to food stimuli among individuals with eating and weight disorders: a systematic review of event-related potentials. Int Rev Psychiatry.

[9] Hiluy JC, David IA, Daquer AF, Duchesne M, Volchan E, Appolinario JC (2021). A systematic review of electrophysiological findings in binge-purge eating disorders: a window into brain dynamics. Front Psychol.

[10] Wolz I, Fagundo AB, Treasure J, Fernández-Aranda F (2015). The processing of food stimuli in abnormal eating: a systematic review of electrophysiology. Eur Eat Disord Rev.

[11] Drobes DJ, Miller EJ, Hillman CH, Bradley MM, Cuthbert BN, Lang PJ (2001). Food deprivation and emotional reactions to food cues: implications for eating disorders. Biol Psychol.

[12] Mauler BI, Hamm AO, Weike AI, Tuschen-Caffier B (2006). Affect regulation and food intake in bulimia nervosa: emotional responding to food cues after deprivation and subsequent eating. J Abnorm Psychol.

[13] Friederich HC, Kumari V, Uher R, Riga M, Schmidt U, Campbell IC (2006). Differential motivational responses to food and pleasurable cues in anorexia and bulimia nervosa: a startle reflex paradigm. Psychol Med.

[14] Schulte EM, Avena NM, Gearhardt AN (2015). Which foods may be addictive? The roles of processing, fat content, and glycemic load. PLoS ONE.

[15] Gearhardt AN (2011). Neural correlates of food addiction. Arch Gen Psychiatry.

[16] Bulik CM, Lawson RH, Carter FA (1996). Salivary reactivity in restrained and unrestrained eaters and women with bulimia nervosa. Appetite.

[17] Fedoroff I, Polivy J, Peter HC (2003). The specificity of restrained versus unrestrained eaters’ responses to food cues: general desire to eat, or craving for the cued food?. Appetite.

[18] Ferrer-García M, Gutiérrez-Maldonado J, Pla-Sanjuanelo J, Vilalta-Abella F, Riva G, Clerici M (2017). A randomised controlled comparison of second-level treatment approaches for treatment-resistant adults with bulimia nervosa and binge eating disorder: assessing the benefits of virtual reality cue exposure therapy: benefits of virtual reality cue exposure therapy. Eur Eat Disorders Rev.

[19] Jansen A, Schyns G, Bongers P, van den Akker K (2016). From lab to clinic: extinction of cued cravings to reduce overeating. Physiol Behav.

[20] Pla-Sanjuanelo J, Ferrer-García M, Gutiérrez-Maldonado J, Riva G, Andreu-Gracia A, Dakanalis A (2015). Identifying specific cues and contexts related to bingeing behavior for the development of effective virtual environments. Appetite.

[21] Jansen A (1998). A learning model of binge eating: cue reactivity and cue exposure. Behav Res Ther.

[22] Eddy KT, Novotny CM, Westen D (2004). Sexuality, personality, and eating disorders. Eat Disord.

[23] Wiederman MW, Pryor T, Morgan CD (1996). The sexual experience of women diagnosed with anorexia nervosa or bulimia nervosa. Int J Eat Disord.

[24] Moreno Dominguez S, Ortega-Roldán B, Rodríguez-Ruiz S. Impulsividad en mujeres con bulimia nerviosa. eduPsykhé. 2009;8(1):63–77.

[25] Katzman MA, Wolchik SA (1984). Bulimia and binge eating in college women: a comparison of personality and behavioral characteristics. J Consult Clin Psychol.

[26] Casper RC (1980). Bulimia: its incidence and clinical importance in patients with anorexia nervosa. Arch Gen Psychiatry.

[27] Raciti M, Hendrick SS (1992). Relationships between eating disorder characteristics and love and sex attitudes. Sex Roles.

[28] Tobin DL, Griffing AS (1996). Coping, sexual abuse, and compensatory behavior. Int J Eat Disord.

[29] Castellini G, Lelli L, Lo Sauro C, Fioravanti G, Vignozzi L, Maggi M (2012). Anorectic and bulimic patients suffer from relevant sexual dysfunctions. J Sex Med.

[30] Pinheiro AP, Raney TJ, Thornton LM, Fichter MM, Berrettini WH, Goldman D, et al. Sexual functioning in women with eating disorders. Int J Eat Disord. 2010;123–9. 10.1002/eat.20671 PMC2820601 19260036

[31] Schupp HT, Schmälzle R, Flaisch T, Weike AI, Hamm AO (2012). Affective picture processing as a function of preceding picture valence: an ERP analysis. Biol Psychol.

[32] Bradley MM, Lang PJ (1994). Measuring emotion: the self-assessment manikin and the semantic differential. J Behav Ther Exp Psychiatry.

[33] Rohde P, Auslander BA, Shaw H, Raineri KM, Gau JM, Stice E (2014). Dissonance-based prevention of eating disorder risk factors in middle school girls: results from two pilot trials: middle school prevention pilots. Int J Eat Disord.

[34] Eisenberg D, Nicklett EJ, Roeder K, Kirz NE (2011). Eating disorder symptoms among college students: prevalence, persistence, correlates, and treatment-seeking. J Am Col Health.

[35] Tavolacci MP, Grigioni S, Richard L, Meyrignac G, Déchelotte P, Ladner J (2015). Eating disorders and associated health risks among university students. J Nutr Educ Behav.

[36] Taylor CB, Bryson S, Luce KH, Cunning D, Doyle AC, Abascal LB (2006). Prevention of eating disorders in at-risk college-age women. Arch Gen Psychiatry.

[37] Miccoli L, Martínez-Fiestas M, Delgado-Rodríguez R, Díaz-Ferrer S, Rodríguez-Ruiz S, Fernández-Santaella MC (2018). Adolescent emotions toward sweet food cues as a function of obesity and risky dieting practices. Food Qual Prefer.

[38] Lang P, Bradley M, Cuthbert B. International affective picture system (IAPS): Affective ratings of pictures and instruction manual. Technical report A-8. University of Florida, Gainesville; 2008.

[39] Wierzba M, Riegel M, Pucz A, Leśniewska Z, Dragan WŁ, Gola M, Jednoróg K, Marchewka A (2015). Erotic subset for the Nencki affective picture system (NAPS ERO): cross-sexual comparison study. Front Psychol.

[40] Guerra P, Campagnoli RR, Vico C, Volchan E, Anllo-Vento L, Vila J (2011). Filial versus romantic love: contributions from peripheral and central electrophysiology. Biol Psychol.

[41] McTeague LM, Lang PJ, Laplante M-C, Cuthbert BN, Shumen JR, Bradley MM (2010). Aversive imagery in posttraumatic stress disorder: trauma recurrence, comorbidity, and physiological reactivity. Biol Psychiatry.

[42] Miccoli L, Delgado R, Rodríguez-Ruiz S, Guerra P, García-Mármol E, Fernández-Santaella MC (2014). Meet OLAF, a good friend of the IAPS! The open library of affective foods: a tool to investigate the emotional impact of food in adolescents. PLoS ONE.

[43] Miccoli L, Delgado R, Guerra P, Versace F, Rodríguez-Ruiz S, Fernández-Santaella MC (2016). Affective pictures and the open library of affective foods (OLAF): tools to investigate emotions toward food in adults. PLoS ONE.

[44] Bradley MM (2007). Brain potentials in perception: PICTURE complexity and emotional arousal. Psychophysiology.

[45] Cook EW (2000). VPM reference manual.

[46] Blumenthal TD, Cuthbert BN, Filion DL, Hackley S, Lipp OV, Van Boxtel A (2005). Committee report: guidelines for human startle eyeblink electromyographic studies. Psychophysiology.

[47] Thelen MH, Farmer J, Wonderlich S, Smith M (1991). A revision of the bulimia test: the BULIT-R. Psychol Assess.

[48] Berrios-Hernandez MN, Rodríguez-Ruiz S, Perez M, Gleaves DH, Maysonet M, Cepeda-Benito A (2007). Cross-cultural assessment of eating disorders: psychometric properties of a Spanish version of the Bulimia test-revised. Eur Eat Disord Rev.

[49] Bradley MM, Lang PJ. Emotion and motivation. In: Cacioppo JT, Tassinary LG, Berntson G, editors. Handbook of psychophysiology. 3th. ed. New York: Cambridge University Press; 2007, p. 581–607. 10.1017/CBO9780511546396.025

[50] Coffey SF, Saladin ME, Drobes DJ, Brady KT, Dansky BS, Kilpatrick DG (2002). Trauma and substance cue reactivity in individuals with comorbid posttraumatic stress disorder and cocaine or alcohol dependence. Drug Dlcohol Depend.

[51] Fairburn CG, Beglin SJ (1994). Assessment of eating disorders: interview or self-report questionnaire?. Int J Eat Disord.

[52] Villarroel AM, Penelo E, Portell M, Raich RM (2011). Screening for eating disorders in undergraduate women: norms and validity of the spanish version of the eating disorder examination questionnaire (EDE-Q). J Psychopathol Behav Assess.

[53] Gearhardt AN, Corbin WR, Brownell KD (2009). Preliminary validation of the yale food addiction scale. Appetite.

[54] Granero R, Jiménez-Murcia S, Gearhardt AN, Agüera Z, Aymamí N, Gómez-Peña M (2018). Validation of the Spanish version of the yale food addiction scale 2.0 (YFAS 2.0) and clinical correlates in a sample of eating disorder, gambling disorder, and healthy control participants. Front Psychiatry.

[55] Snaith RP, Hamilton M, Morley S, Humayan A, Hargreaves D, Trigwell P (1995). A Scale for the assessment of hedonic tone the snaith–hamilton pleasure scale. Br J Psychiatry.

[56] Fresán A, Berlanga C (2013). Traducción al español y validación de la Escala de Placer Snaith-Hamilton para Anhedonia (shaps). Actas Esp Psiquiatr.

[57] Cooper PJ, Taylor MJ, Cooper Z, Fairbum CG (1987). The development and validation of the body shape questionnaire. Int J Eat Disord.

[58] Raich RM, Mora M, Soler A, Ávila C, Clos I, Zapater L (1996). Adaptación de un instrumento de evaluación de la insatisfacción corporal. Clín Salud.

[59] Balaban MT, Losito B, Simons RF, Graham FK (1986). Off-line latency and amplitude scoring of the human reflex eye blink with Fortran IV. Psychophysiology.

[60] Racine SE, Hebert KR, Benning SD (2018). Emotional reactivity and appraisal of food in relation to eating disorder cognitions and behaviours: evidence to support the motivational conflict hypothesis. Eur Eat Disord Rev.

[61] Racine SE, Suissa-Rocheleau L, Martin SJ, Benning SD (2021). Implicit and explicit motivational responses to high- and low-calorie food in women with disordered eating. Int J Psychophysiol.

[62] Schnepper R, Georgii C, Eichin K, Arend A-K, Wilhelm FH, Vögele C (2020). Fight, flight, – or grab a bite! Trait emotional and restrained eating style predicts food cue responding under negative emotions. Front Behav Neurosci.

[63] Weygandt M, Schaefer A, Schienle A, Haynes J (2012). Diagnosing different binge-eating disorders based on reward-related brain activation patterns. Hum Brain Mapp.

[64] Yilmaz Z, Gottfredson NC, Zerwas SC, Bulik CM, Micali N (2019). Developmental premorbid body mass index trajectories of adolescents with eating disorders in a longitudinal population cohort. J Am Acad Child Adolesc Psychiatr.

[65] Stice E, Marti CN, Durant S (2011). Risk factors for onset of eating disorders: evidence of multiple risk pathways from an 8-year prospective study. Behav Res Ther.

[66] Meule A, von Rezori V, Blechert J (2014). Food addiction and Bulimia Nervosa. Eur Eat Disord Rev.

[67] Pursey K, Stanwell P, Gearhardt A, Collins C, Burrows T (2014). The Prevalence of food addiction as assessed by the yale food addiction scale: a systematic review. Nutrients.

[68] Faul F, Erdfelder E, Buchner A, Lang AG (2009). Statistical power analyses using G* Power 3.1: tests for correlation and regression analyses. Behav Res Methods.

[69] Suissa-Rocheleau L, Benning SD, Racine SE (2019). Associations between self-report and physiological measures of emotional reactions to food among women with disordered eating. Int J Psychophysiol.

[70] Stockburger J, Schmälzle R, Flaisch T, Bublatzky F, Schupp HT (2009). The impact of hunger on food cue processing: an event-related brain potential study. Neuroimage.

[71] Ster Wallin G, Norring C, Holmgren S (1994). Binge eating versus nonpurged eating in bulimics: is there a carbohydrate craving after all?. Acta Psychiatr Scand.

[72] Castellini G, Lelli L, Ricca V, Maggi M (2016). Sexuality in eating disorders patients: etiological factors, sexual dysfunction and identity issues. A systematic review. Horm Mol Biol Clin Investig.

[73] Don Morgan C, Wederman MW, Pryor TL (1995). Sexual functioning and attitudes of eating-disordered women: a follow-up study. J Sex Marital Ther.

[74] Rothschild BS, Fagan PJ, Woodall C, Andersen AE (1991). Sexual functioning of female eating-disordered patients. Int J Eat Disord.

[75] Westen D, Harnden-Fischer J (2001). Personality profiles in eating disorders: Rethinking the distinction between axis I and axis II. AJP.

[76] Blechert J, Ansorge U, Tuschen-Caffier B (2010). A body-related dot-probe task reveals distinct attentional patterns for bulimia nervosa and anorexia nervosa. J Abnorm Psychol.

[77] Friederich HC, Uher R, Brooks S, Giampietro V, Brammer M, Williams SCR, Herzog W, Treasure J, Campbell IC (2007). I'm not as slim as that girl: Neural bases of body shape self-comparison to media images. Neuroimage.

[78] Friederich HC, Brooks S, Uher R, Campbell IC, Giampietro V, Brammer M, Williams SCR, Herzog W, Treasure J (2010). Neural correlates of body dissatisfaction in anorexia nervosa. Neuropsychologia.

[79] Fairburn C, Cooper Z, O'Connor M. Eating disorder examination (Edition 16.0D). In: Fairburn C, editor. Cognitive behavior therapy and eating disorders. New York: Guilford Press; 2008:265–308.

[80] Kühnpast N, Gramann K, Pollatos O (2012). Electrophysiologic evidence for multilevel deficits in emotional face processing in patients with bulimia nervosa. Psychosom Med.

[81] Merlotti E, Mucci A, Volpe U, Montefusco V, Monteleone P, Bucci P, Galderisi S (2013). Impulsiveness in patients with bulimia nervosa: electrophysiological evidence of reduced inhibitory control. Neuropsychobiology.

[82] Otagaki Y, Tohoda Y, Osada M, Horiguchi J, Yamawaki S (1998). Prolonged P300 latency in eating disorders. Neuropsychobiology.

