# Supplementary material for: Emotional reactivity to binge food and erotic cues in women with bulimia nervosa symptoms
Source: J Eat Disord. 2021 Sep 28;9:120. doi: 10.1186/s40337-021-00475-9 (PMC8479974; doi:10.1186/s40337-021-00475-9)
Supplement: Supplementary file 1 — Additional file 1. Extra analyses to examine the influence of food contents on emotional reactivity and the influence of female bodies on valence results. [file 40337_2021_475_MOESM1_ESM.docx]

**Emotional Reactivity to Binge Food and Erotic Cues in Women with Bulimia Nervosa Symptoms**

Isabel Hernández-Rivero, Jens Blechert, Laura Miccoli, Katharina Naomi Eichin, M. Carmen Fernández-Santaella, Rafael Delgado-Rodríguez

**Additional file 1**

*Influence of food contents on emotional reactivity*

To examine if food contents might affect emotional reactivity in the current study, we performed extra-analyses comparing women that mostly selected high-calorie sweet food (>=66.7% of pictures were sweet, n=9 subjects) with women whose binge foods were mostly fat (>=66.7% of pictures were fat, n=49 subjects). The mixed ANOVAs, 2 (Group) x 4 (Picture Category), did not yield significant Group x Picture Category interactions for any psychophysiological (blink, corrugator, and zygomatic) and subjective (valence, arousal, and dominance) measure.

*Influence of female bodies on valence results*

Given that body cues are relevant for women with eating-related problems and they are therefore differently processed in this sample (e.g., 1,2), we performed extra analyses to examine the possible impact of female bodies on the current valence result (i.e., the association between valence reactions to erotic cues and bulimic symptoms). For that, we first grouped erotic pictures in two groups; those depicting the whole –naked– female body or body parts (excluding head) were grouped together (n=19), and those where no female body parts appear or women were completely dressed (n=5). Afterwards, we performed a repeated ANCOVA (including valence to both Picture Categories as within-group variable and BULIT-R scores as covariate), which yielded only a significant main effect of BULIT-R (F(1,73)=4.704, *p*<.05, partial η^2^=.061), indicating that BULIT-R scores influence valence ratings regardless the erotic picture category (the higher BULIT-R scores are associated with higher valence).

References

1. Friederich HC, Kumari V, Uher R, Riga M, Schmidt U, Campbell IC, et al. Differential motivational responses to food and pleasurable cues in anorexia and bulimia nervosa: a startle reflex paradigm. Psychol Med. 2006;36(9):1327-35.
2. Uher, R., Murphy, T., Friederich, H. C., Dalgleish, T., Brammer, M. J., Giampietro, V., ... & Treasure, J. (2005). Functional neuroanatomy of body shape perception in healthy and eating-disordered women. *Biological psychiatry*, *58*(12), 990-997.
